# Applying the Taguchi Method to River Water Pollution Remediation Strategy Optimization

**DOI:** 10.3390/ijerph110404108

**Published:** 2014-04-15

**Authors:** Tsung-Ming Yang, Nien-Sheng Hsu, Chih-Chiang Chiu, Hsin-Ju Wang

**Affiliations:** 1 Department of Civil Engineering, National Taiwan University, Taipei 10617, Taiwan; E-Mail: ak8687@ntpc.gov.tw; 2 Wastewater Sewerage System Engineering Division, Water Resources Department, New Taipei City government 234, Taiwan; E-Mail: Cchiang719@gmail.com; 3 MWH Americas Inc., Taiwan Branch 106, Taiwan; E-Mail: leo.wang@mwhglobal.com

**Keywords:** water pollution remediation, optimization, Taguchi method, orthogonal arrays

## Abstract

Optimization methods usually obtain the travel direction of the solution by substituting the solutions into the objective function. However, if the solution space is too large, this search method may be time consuming. In order to address this problem, this study incorporated the Taguchi method into the solution space search process of the optimization method, and used the characteristics of the Taguchi method to sequence the effects of the variation of decision variables on the system. Based on the level of effect, this study determined the impact factor of decision variables and the optimal solution for the model. The integration of the Taguchi method and the solution optimization method successfully obtained the optimal solution of the optimization problem, while significantly reducing the solution computing time and enhancing the river water quality. The results suggested that the basin with the greatest water quality improvement effectiveness is the Dahan River. Under the optimal strategy of this study, the severe pollution length was reduced from 18 km to 5 km.

## 1. Introduction

River basin pollution can be classified into four types according to the sources, which are domestic sewage, livestock wastewater, industrial wastewater, and non-point source pollution. If the river flows through a large metropolitan area, the main pollution source will be the domestic sewage. To remedy the domestic sewage pollution, the most effective strategy is the construction of sewerage systems. However, the sewerage system construction period is long, and domestic sewage continues to be discharged without treatment until the entire system is completely constructed. Therefore, besides building the sewerage system, the remediation strategy should also concern the water pollution treatment problem during the construction period. 

This study aims to use the verified water quality model, from tens of water pollution remediation strategies or facilities, and integrate a method that can quickly determine the optimal remediation measure combination, in order to reduce the number of operations, and obtain the optimal combination of remediation strategy operations. Previous studies have used the optimization model to obtain the optimal solution. However, in practice, tens of combinations of water pollution remediation measures can result in an enormous solution space. Moreover, the calculation of water quality models is time consuming, thus leading to poor efficiency and infeasibility for field application due to the long solving process. Therefore, this study applies the Taguchi method, which has high efficiency in solving problems, in the optimization model, in order to obtain the influence coefficient of decision variable combinations on the reached targets. Then, the optimal strategy combination is determined according to the influence coefficient. This study takes the Danshui River basin in Taiwan for case analysis. The research process includes four parts: collection of basic data and river remediation strategies, construction of optimization model, input of the Taguchi method, and result analysis.

## 2. Research Area

**Figure 1:**
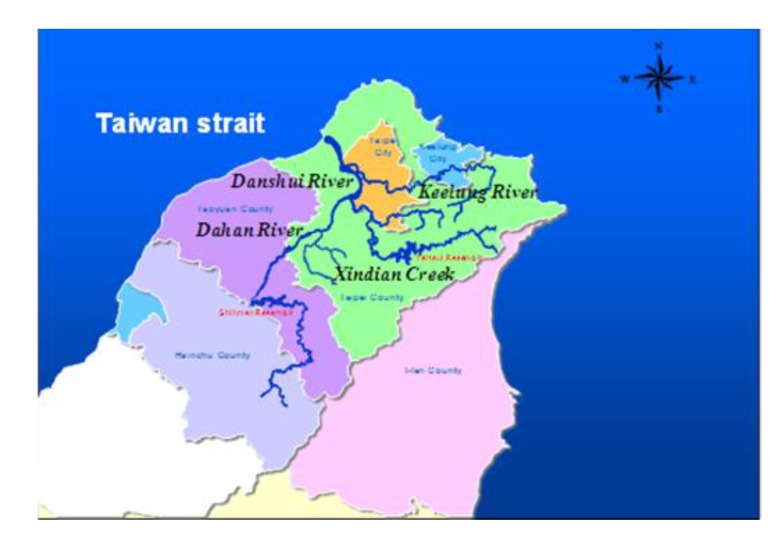
Danshui River basin range.

**Figure 2:**
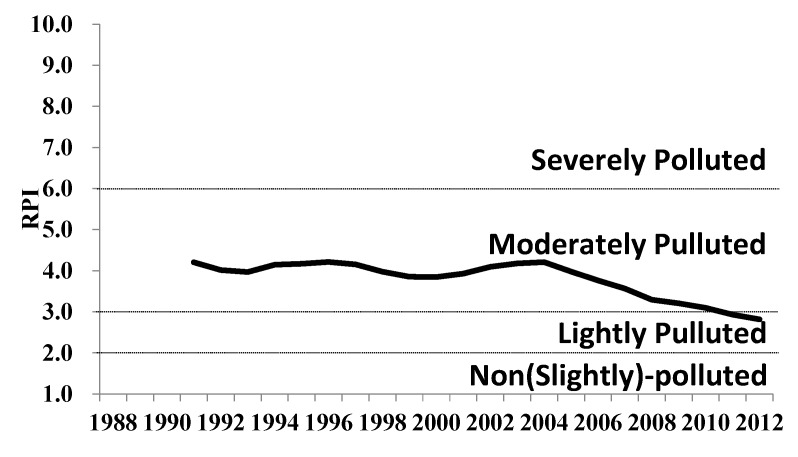
RPI trend map of Danshui River basin (RPI: River Pollution Index).

The Danshui River is the third longest river in Taiwan, running for 158 km and draining a watershed of approximately 2,726 km^2^ (Figure 1). Because the Danshui River has had a long history of water pollution from municipal and industrial wastewaters, the government’s cleanup effort focused chiefly on rendering the polluted river odorless and ensuring that the ambient water has sufficient dissolved oxygen (DO) to sustain aquatic life, while also breaking down biological oxygen demand (BOD). The government uses a River Pollution Index based on the DO, BOD, ammonia, and suspended solids levels. Prior to 2002, however, Taiwan’s previous effort to curb water pollution progressed slowly. In 2012, the government switched its focus from building sewerage systems with central collection and treatment processes to treating wastewaters on site along the river’s upstream and midstream sections. Compared with conventional systems, which require significant time and money to build, onsite treatment systems, through the construction of wetlands and gravel beds in the river, can yield efficient cleanup at a much faster pace [1,2,3,4,5,6,7]. As shown in Figure 2, the overall water quality trend of Danshui River has changed significantly since 2003, and its condition has shifted towards light pollution.

**Figure 3:**
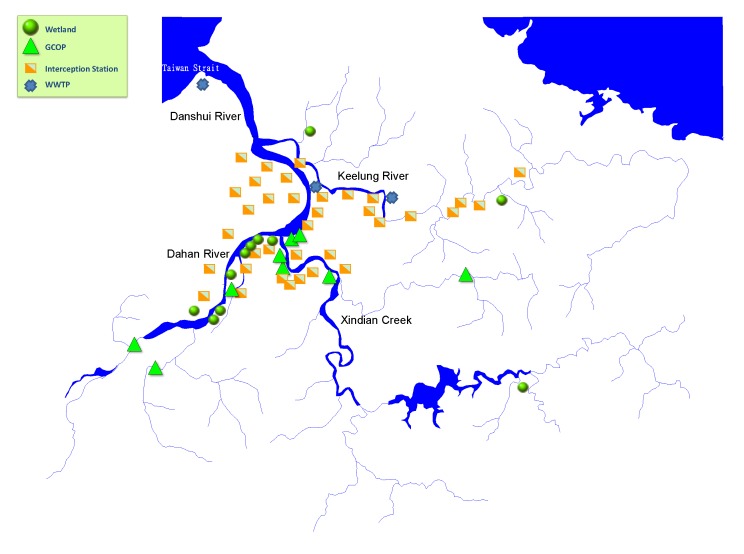
Danshui River basin sewage treatment installation distribution diagram.

According to the key remediation measures, the rate of household connection in the cities around the Danshui River basin, by the end of 2012, is 70.4% in Taipei City and 49.5% in New Taipei City. There are three main sewage treatment plants, 35 interception stations and 21 onsite treatment facilities in the whole basin. The locations are shown in Figure 3.

The total designed capacity and onsite treatment capacity of interception stations in the Danshui River basin, the total household connection capacity, and the total treatment capacity of sewage treatment plants in 2012 are shown in Table 1. As seen, the total designed interception capacity and total household connection capacity of interception stations in the Danshui River basin have exceeded the treatment capacity of sewage treatment plants. Therefore, it is infeasible to operate all the interception stations. Secondly, in the drainage channels of the basin, a part of sewage network fails to meet or requires emergency treatment, and onsite treatment facilities are implemented. The total capacity and the total interception capacity have already exceeded the load capacity of sewage plants. Therefore, how to determine the start/stop of interception and onsite treatment facilities is a challenge to the present water pollution remediation in Danshui River basin.

**Table 1:** Comparison of treatment facility and sewage plant capacities.

Item	Flow Rate (m^3^/day)
Total Interception Capacity	2,320,184
Total Onsite Treatment Capacity	247,054
Household connection capacity in 2012	1,174,621
Waste water treatment capacity	1,970,000

## 3. Methodology

In practice, the remediation of river water pollution first sets the objectives and period, and then analyzes the effect of remediation strategy, in order to select the optimal strategy combination for attaining the objectives.

In the optimization model solving process, the solution space formed by the decision variables is too large, and the water quality model computation is time consuming, resulting in poor model solving efficiency. Among studies on connecting optimization to simulation models, the speed of optimal solution search is the key factor influencing the overall computing efficiency. In order to improve the computing efficiency, this study uses the Taguchi method and regards the optimization model as a system. The decision variables are experimental parameters, and the objective function is system function. The experimental parameters are changed to obtain the variable parameters for overall function variation, thus accelerating the computational speed for the optimal combination of decision variables, and facilitating the decision-making on the optimal remediation strategy. 

This research process includes: (1) determining the key features among domestic sewage—sewage treatment strategy—rivers; (2) developing a river water quality model to simulate river water quality under different strategies; (3) applying the Taguchi method to build an optimization model for selecting optimal strategy. The steps are described below:

### 3.1. Sewage Treatment Correlation and Water Quality Modeling

**Figure 4:**
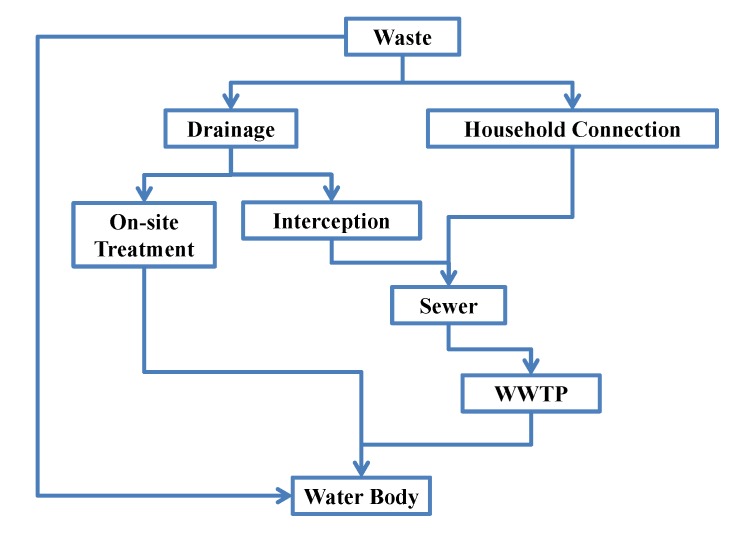
Schematic diagram of sewage treatment strategy.

The sewer policy of Taiwan is based on a rain and sewage diversion system. Before the household connection is completed, the domestic sewage is discharged to the rivers by the rainwater collection system. On sunny days, the high concentration domestic sewage discharged into rivers in this way deteriorates the river water quality. In order to avoid the direct discharge of high concentration sewage, the current improvement measures, besides promoting household connection, include: (1) special pipes at the drainage end intercepts sewage for onsite treatment before being discharged; (2) the interception facilities intercept the sewage into the main sewer with remaining capacity, and the sewage is delivered to the downstream sewage treatment plant to drain after treatment. The overall regional sewage treatment strategy, including the above two remediation strategies, is illustrated in Figure 4.

In Taiwan, there is no domestically developed river water quality model, and all the above models are adopted from foreign experiences. The commonly used water quality models include WASP, QUAL2K, and SWMM. In terms of the Danshui River basin, in order to unify the water quality model tool, the Environmental Protection Administration (EPA) adopted WASP as the main model for simulating the Danshui River water quality. Since then, numerous studies have used WASP as the tool for Danshui River basin. This study uses the Danshui River model built by Chen *et al.* [8,9] as the main simulation tool, and includes the completed sewage treatment related facilities, domestic sewage connection, interception into main sewer, onsite treatment facilities, and direct discharge into river. The situation simulation and effect evaluation under various strategies are discussed [1,2,3,4,5,6,7].

### 3.2. Taguchi Method

The Taguchi method is a quality engineering proposed by Genichi Taguchi. The method includes off-line and on-line quality engineering and MTS (Mahalanobis-Taguchi System). This study uses off-line quality engineering method, which aims to improve R&D, design and production technology for solving optimization problem. The purposes are to obtain the optimal improvement measures, including functional evaluation of quality, determination of specifications (tolerance), parameter design and tolerance design [10].

Orthogonal arrays and S/N ratios are two main components of the Taguchi method. An orthogonal array is used to reduce testing time/cost. If an experiment has 15 control factors with two levels, all possible *n* = 215 = 32,768 mixtures are required to test for assessing the optimal mixture by using a full factorial design of experiment. After using the orthogonal array L16215, only 16 mixtures are required to estimate the optimal mixture *vs.* the optimal determination via the full factorial design of experiment. To significantly reduce the number of tests, while still gaining significant insight into the important factors and optimal settings, Taguchi recommended the use of eighteen basic orthogonal fractional factorial arrays, known as the standard orthogonal arrays. Taguchi treated the S/N ratio as a single indicator that considers the statistical characteristics of results to determine the importance of the factors under study. The S/N ratio can be categorized into three types as follows. Selection of the appropriate S/N ratio depends on the features of responses.

1. The smaller-the-better (STB) type:

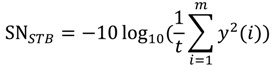
(1)
2. The larger-the-better (LTB) type:

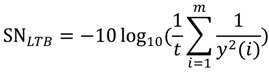
(2)
3. The nominal-the-better (NTB) type:

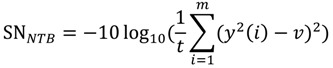
(3)

where *m*, *y*(*i*), and m are the number of mixtures, the testing result of the i-th test, and the target value of the response, respectively [11].

### 3.3. Construction and Solving of an Optimization Model

**Figure 5:**
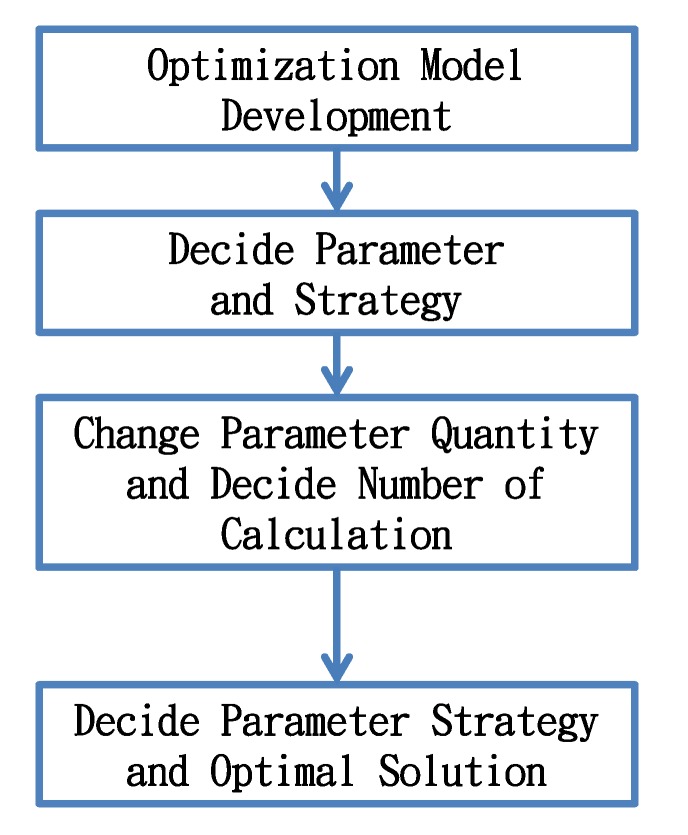
Solving process of this study.

The solving process proposed by this study is to build the optimization model first, and then determine the number of system parameters and level. The level of parameters is changed according to the orthogonal arrays of the Taguchi method, and the system is simulated repeatedly. Finally, the sensitivity of parameters to the system and the optimal solution of system are obtained. The solving process is shown in Figure 5.

#### 3.3.1. Optimization Model Setting

The objective function of this optimization model is set as the minimum average RPI of the entire Danshui River basin; the purpose of which is to optimize the remediation strategy for the lower river pollution level. The decision variables are the strategies of various treatment facilities, and the constraints are the limitations of treatment facility strategies (the total interception capacity of interception stations along main sewer does not exceed the remaining capacity of the main sewer and the onsite treatment capacity does not exceed the designed capacity). The amount of sewage received by various treatment facilities is the amount of domestic sewage in the corresponding sewage collection area. The total flow of main sewer does not exceed the treatment capacity of sewage treatment plant. The basin pollution concentration is the computing result of WASP water quality model. The optimization model is expressed as follows:

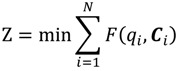
(4)

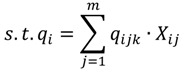
(5)

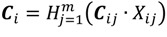
(6)

*q_ijk_* = *L_ij_* · *S_ij_* (7)

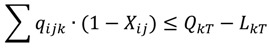
(8)

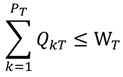
(9)

Equation (4) is the objective function Z, representing basin-wide minimum average PRI, *i* is the water quality model computation node, N is the number of nodes of water quality model, *F*(*q_i_*,*C_i_*) is the water quality model simulation result, displayed as basin-wide average RPI. The *q_i_* and *C_i_* are the flow rate and water quality at the i-th computation node.

Equations (5) and (6) show the flow rate and water quality at the *i*-th computation node and the effect of the facility strategy in the computation node on the node, where *q_ijk_* is the water quantity discharged to the river by the *j*-th facility of the *k*-th main pipe system in the *i*-th node, *X_ij_* is the facility operating strategy, as well as the decision variable of this study; *C_ij_* is the quality of water discharged to the river by the *j*-th facility in the *i*-th node (given constant); if *k* = 0, it is onsite treatment facility.

Equation (7) is the amount of domestic sewage of the *j*-th facility in the *i*-th node, where *L_ij_* is the sewage collection area corresponding to the *j*-th facility in the *i*-th computation node; and *S_ij_* is the sewage delivery rate of the sewage collection area (given constant).

In Equation (8), Σ*q_ijk_* · (1 - *X_ij_*) is the flow rate into the *k*-th sewer pipe through the *j*-th interception station of the *i*-th computation node; *Q_kT_* - *L_kT_* is the remaining capacity of the *k*-th sewer pipe of the *T*-th sewage treatment plant (given constant).

In Equation (9), 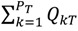 is the total flow into the *k*-th sewer pipe of the *T*-th sewage treatment plant; W_T_ is the treatment capacity of the *T*-th sewage treatment plant (given constant).

#### 3.3.2. Determine System Parameters and Strategies

The decision variable of this study is the facility operating strategy *X_ij_*, *i.e.*, the start-stop condition of the facility. To determine the number of system parameters, this study considers the interception stations and onsite treatment facilities with high operating flexibility in the Danshui River basin as system parameters. There are 31 treatment facilities determined as system parameters after comparing the treatment facility operating data. In addition, in order to reduce the solution space of the optimization model, each system parameter has two operating strategies *X_ij_* (*i.e.*, 0% and 100%).

#### 3.3.3. Change Parameters, Repeat Simulating System

When the number of system parameters and operating strategies are determined, the number of repeated simulations and the values of parameters in each simulation can be determined according to the orthogonal array of the Taguchi method. This study selects 31 system parameters and each parameter has two operating strategies. The optimum parameter combination can be obtained according to the simulation result of only 32 repeated system simulations. In comparison to general optimization, which needs to find the optimal solution among 2^31^ = 2,147,483,648 permutations, the Taguchi method can shorten the computation time greatly. The 31 system parameters selected by this study are indicated as codes A,B~AE, while 1 means that the operating strategy is 100%, 2 means that the operating strategy is 0%. For example, the value of parameter A in the first strategy combination is 1, meaning that the operating strategy of parameter A in the strategy combination is 100%, and so forth. The parameters in various strategy permutations are shown in Table 2.

#### 3.3.4. Determine Parameter Impact Factor and Optimal Solution of System

**Figure 6:**
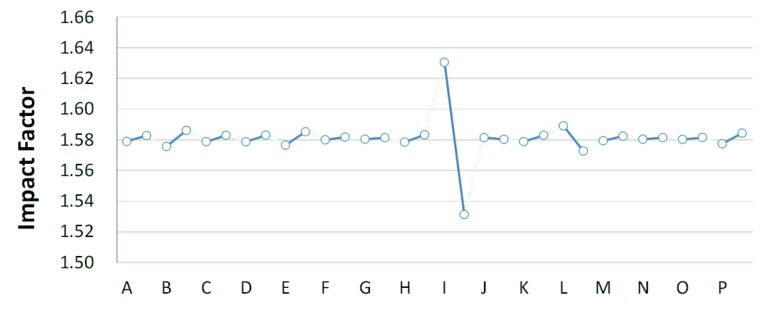
Danshui River water pollution remediation strategy simulation results- Dahan River BOD_5_ concentration.

**Figure 7:**
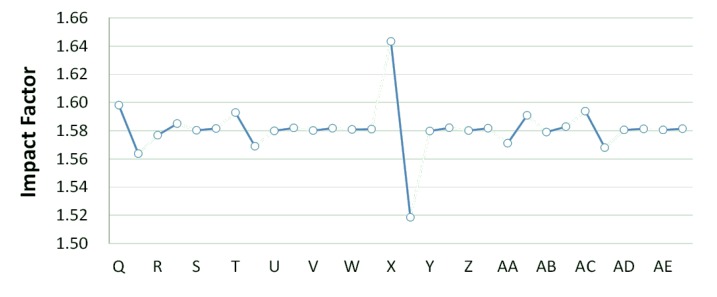
Danshui River water pollution remediation strategy simulation results- Dahan River BOD_5_ concentration.

The basin-wide average water quality of each simulation is determined after 32 simulations. The basin-wide average water quality under the same operating strategy value of the same facility is calculated. Finally, the values under different strategies are subtracted to obtain the influence coefficient of the facility (system parameters), as shown in Figure 6 and Figure 7, and Table 3. The influence coefficient in this study represents the influence of treatment facility changing strategy on basin-wide water quality. Therefore, a larger influence coefficient indicates greater improvement on the basin-wide water quality. This study reviews the sequence of influence coefficients of facilities on river water quality and the remaining capacity in their locations. If the remaining capacity is sufficient, the facility is switched on (100%), otherwise it is switched off (0%).

**Table 2:** System simulation parameter permutations.

Strategy Parameter	No. 1	No. 2	No. 3	No. 4	No. 5	No. 6	No. 7	No. 8	No. 9	No. 10	No. 11	No. 12	No. 13	No. 14	No. 15	No. 16	No. 17	No. 18	No. 19	No. 20	No. 21	No. 22	No. 23	No. 24	No. 25	No. 26	No. 27	No. 28	No. 29	No. 30	No. 31	No. 32
**A**	**1**	**1**	**1**	**1**	**1**	**1**	**1**	**1**	**1**	**1**	**1**	**1**	**1**	**1**	**1**	**1**	**2**	**2**	**2**	**2**	**2**	**2**	**2**	**2**	**2**	**2**	**2**	**2**	**2**	**2**	**2**	**2**
**B**	**1**	**1**	**1**	**1**	**1**	**1**	**1**	**1**	**2**	**2**	**2**	**2**	**2**	**2**	**2**	**2**	**1**	**1**	**1**	**1**	**1**	**1**	**1**	**1**	**2**	**2**	**2**	**2**	**2**	**2**	**2**	**2**
**C**	**1**	**1**	**1**	**1**	**1**	**1**	**1**	**1**	**2**	**2**	**2**	**2**	**2**	**2**	**2**	**2**	**2**	**2**	**2**	**2**	**2**	**2**	**2**	**2**	**1**	**1**	**1**	**1**	**1**	**1**	**1**	**1**
**D**	**1**	**1**	**1**	**1**	**2**	**2**	**2**	**2**	**1**	**1**	**1**	**1**	**2**	**2**	**2**	**2**	**1**	**1**	**1**	**1**	**2**	**2**	**2**	**2**	**1**	**1**	**1**	**1**	**2**	**2**	**2**	**2**
**E**	**1**	**1**	**1**	**1**	**2**	**2**	**2**	**2**	**1**	**1**	**1**	**1**	**2**	**2**	**2**	**2**	**2**	**2**	**2**	**2**	**1**	**1**	**1**	**1**	**2**	**2**	**2**	**2**	**1**	**1**	**1**	**1**
**F**	**1**	**1**	**1**	**1**	**2**	**2**	**2**	**2**	**2**	**2**	**2**	**2**	**1**	**1**	**1**	**1**	**1**	**1**	**1**	**1**	**2**	**2**	**2**	**2**	**2**	**2**	**2**	**2**	**1**	**1**	**1**	**1**
**G**	**1**	**1**	**1**	**1**	**2**	**2**	**2**	**2**	**2**	**2**	**2**	**2**	**1**	**1**	**1**	**1**	**2**	**2**	**2**	**2**	**1**	**1**	**1**	**1**	**1**	**1**	**1**	**1**	**2**	**2**	**2**	**2**
**H**	**1**	**1**	**2**	**2**	**1**	**1**	**2**	**2**	**1**	**1**	**2**	**2**	**1**	**1**	**2**	**2**	**1**	**1**	**2**	**2**	**1**	**1**	**2**	**2**	**1**	**1**	**2**	**2**	**1**	**1**	**2**	**2**
**I**	**1**	**1**	**2**	**2**	**1**	**1**	**2**	**2**	**1**	**1**	**2**	**2**	**1**	**1**	**2**	**2**	**2**	**2**	**1**	**1**	**2**	**2**	**1**	**1**	**2**	**2**	**1**	**1**	**2**	**2**	**1**	**1**
**J**	**1**	**1**	**2**	**2**	**1**	**1**	**2**	**2**	**2**	**2**	**1**	**1**	**2**	**2**	**1**	**1**	**1**	**1**	**2**	**2**	**1**	**1**	**2**	**2**	**2**	**2**	**1**	**1**	**2**	**2**	**1**	**1**
**K**	**1**	**1**	**2**	**2**	**1**	**1**	**2**	**2**	**2**	**2**	**1**	**1**	**2**	**2**	**1**	**1**	**2**	**2**	**1**	**1**	**2**	**2**	**1**	**1**	**1**	**1**	**2**	**2**	**1**	**1**	**2**	**2**
**L**	**1**	**1**	**2**	**2**	**2**	**2**	**1**	**1**	**1**	**1**	**2**	**2**	**2**	**2**	**1**	**1**	**1**	**1**	**2**	**2**	**2**	**2**	**1**	**1**	**1**	**1**	**2**	**2**	**2**	**2**	**1**	**1**
**M**	**1**	**1**	**2**	**2**	**2**	**2**	**1**	**1**	**1**	**1**	**2**	**2**	**2**	**2**	**1**	**1**	**2**	**2**	**1**	**1**	**1**	**1**	**2**	**2**	**2**	**2**	**1**	**1**	**1**	**1**	**2**	**2**
**N**	**1**	**1**	**2**	**2**	**2**	**2**	**1**	**1**	**2**	**2**	**1**	**1**	**1**	**1**	**2**	**2**	**1**	**1**	**2**	**2**	**2**	**2**	**1**	**1**	**2**	**2**	**1**	**1**	**1**	**1**	**2**	**2**
**O**	**1**	**1**	**2**	**2**	**2**	**2**	**1**	**1**	**2**	**2**	**1**	**1**	**1**	**1**	**2**	**2**	**2**	**2**	**1**	**1**	**1**	**1**	**2**	**2**	**1**	**1**	**2**	**2**	**2**	**2**	**1**	**1**
**P**	**1**	**2**	**1**	**2**	**1**	**2**	**1**	**2**	**1**	**2**	**1**	**2**	**1**	**2**	**1**	**2**	**1**	**2**	**1**	**2**	**1**	**2**	**1**	**2**	**1**	**2**	**1**	**2**	**1**	**2**	**1**	**2**
**Q**	**1**	**2**	**1**	**2**	**1**	**2**	**1**	**2**	**1**	**2**	**1**	**2**	**1**	**2**	**1**	**2**	**2**	**1**	**2**	**1**	**2**	**1**	**2**	**1**	**2**	**1**	**2**	**1**	**2**	**1**	**2**	**1**
**R**	**1**	**2**	**1**	**2**	**1**	**2**	**1**	**2**	**2**	**1**	**2**	**1**	**2**	**1**	**2**	**1**	**1**	**2**	**1**	**2**	**1**	**2**	**1**	**2**	**2**	**1**	**2**	**1**	**2**	**1**	**2**	**1**
**S**	**1**	**2**	**1**	**2**	**1**	**2**	**1**	**2**	**2**	**1**	**2**	**1**	**2**	**1**	**2**	**1**	**2**	**1**	**2**	**1**	**2**	**1**	**2**	**1**	**1**	**2**	**1**	**2**	**1**	**2**	**1**	**2**
**T**	**1**	**2**	**1**	**2**	**2**	**1**	**2**	**1**	**1**	**2**	**1**	**2**	**2**	**1**	**2**	**1**	**1**	**2**	**1**	**2**	**2**	**1**	**2**	**1**	**1**	**2**	**1**	**2**	**2**	**1**	**2**	**1**
**U**	**1**	**2**	**1**	**2**	**2**	**1**	**2**	**1**	**1**	**2**	**1**	**2**	**2**	**1**	**2**	**1**	**2**	**1**	**2**	**1**	**1**	**2**	**1**	**2**	**2**	**1**	**2**	**1**	**1**	**2**	**1**	**2**
**V**	**1**	**2**	**1**	**2**	**2**	**1**	**2**	**1**	**2**	**1**	**2**	**1**	**1**	**2**	**1**	**2**	**1**	**2**	**1**	**2**	**2**	**1**	**2**	**1**	**2**	**1**	**2**	**1**	**1**	**2**	**1**	**2**
**W**	**1**	**2**	**1**	**2**	**2**	**1**	**2**	**1**	**2**	**1**	**2**	**1**	**1**	**2**	**1**	**2**	**2**	**1**	**2**	**1**	**1**	**2**	**1**	**2**	**1**	**2**	**1**	**2**	**2**	**1**	**2**	**1**
**X**	**1**	**2**	**2**	**1**	**1**	**2**	**2**	**1**	**1**	**2**	**2**	**1**	**1**	**2**	**2**	**1**	**1**	**2**	**2**	**1**	**1**	**2**	**2**	**1**	**1**	**2**	**2**	**1**	**1**	**2**	**2**	**1**
**Y**	**1**	**2**	**2**	**1**	**1**	**2**	**2**	**1**	**1**	**2**	**2**	**1**	**1**	**2**	**2**	**1**	**2**	**1**	**1**	**2**	**2**	**1**	**1**	**2**	**2**	**1**	**1**	**2**	**2**	**1**	**1**	**2**
**Z**	**1**	**2**	**2**	**1**	**1**	**2**	**2**	**1**	**2**	**1**	**1**	**2**	**2**	**1**	**1**	**2**	**1**	**2**	**2**	**1**	**1**	**2**	**2**	**1**	**2**	**1**	**1**	**2**	**2**	**1**	**1**	**2**
**AA**	**1**	**2**	**2**	**1**	**1**	**2**	**2**	**1**	**2**	**1**	**1**	**2**	**2**	**1**	**1**	**2**	**2**	**1**	**1**	**2**	**2**	**1**	**1**	**2**	**1**	**2**	**2**	**1**	**1**	**2**	**2**	**1**
**AB**	**1**	**2**	**2**	**1**	**2**	**1**	**1**	**2**	**1**	**2**	**2**	**1**	**2**	**1**	**1**	**2**	**1**	**2**	**2**	**1**	**2**	**1**	**1**	**2**	**1**	**2**	**2**	**1**	**2**	**1**	**1**	**2**
**AC**	**1**	**2**	**2**	**1**	**2**	**1**	**1**	**2**	**1**	**2**	**2**	**1**	**2**	**1**	**1**	**2**	**2**	**1**	**1**	**2**	**1**	**2**	**2**	**1**	**2**	**1**	**1**	**2**	**1**	**2**	**2**	**1**
**AD**	**1**	**2**	**2**	**1**	**2**	**1**	**1**	**2**	**2**	**1**	**1**	**2**	**1**	**2**	**2**	**1**	**1**	**2**	**2**	**1**	**2**	**1**	**1**	**2**	**2**	**1**	**1**	**2**	**1**	**2**	**2**	**1**
**AE**	**1**	**2**	**2**	**1**	**2**	**1**	**1**	**2**	**2**	**1**	**1**	**2**	**1**	**2**	**2**	**1**	**2**	**1**	**1**	**2**	**1**	**2**	**2**	**1**	**1**	**2**	**2**	**1**	**2**	**1**	**1**	**2**

**Table 3:** System impact factors.

Rank	Impact Factor	Facility	Rank	Impact Factor	Facility
1	0.1248	X	17	0.0037	A
2	0.0993	I	18	0.0030	M
3	0.0343	Q	19	0.0023	Y
4	0.0258	AC	20	0.0020	U
5	0.0238	T	21	0.0018	F
6	0.0198	AA	22	0.0017	V
7	0.0164	L	23	0.0016	Z
8	0.0104	B	24	0.0012	S
9	0.0086	E	25	0.0012	O
10	0.0082	R	26	0.0011	J
11	0.0069	P	27	0.0011	G
12	0.0047	H	28	0.0010	N
13	0.0043	D	29	0.0009	AE
14	0.0041	C	30	0.0008	AD
15	0.0040	K	31	0.0002	W
16	0.0038	AB			

## 4. Results

**Figure 8:**
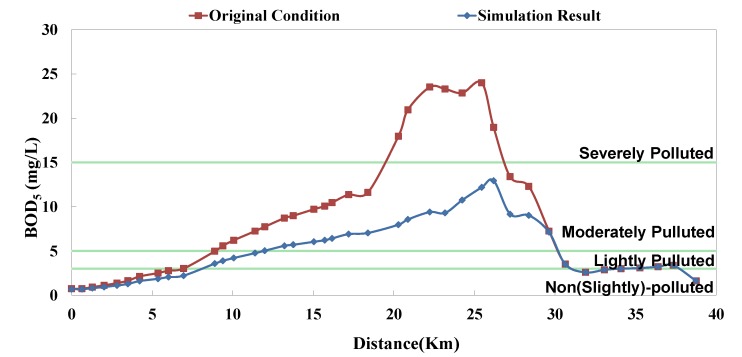
Danshui River water pollution remediation strategy simulation results- Dahan River BOD_5_ concentration.

**Figure 9:**
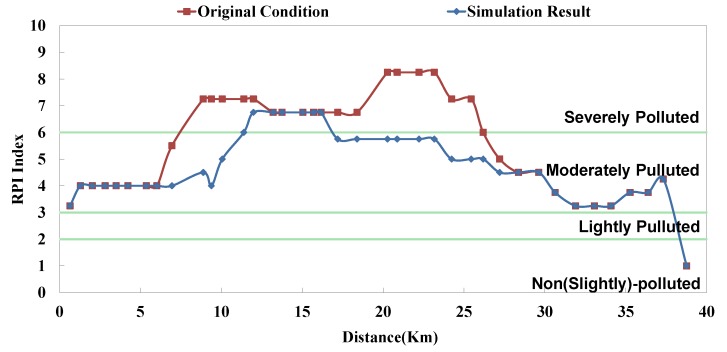
Danshui River water pollution remediation strategy simulation results- Dahan River RPI.

**Figure 10:**
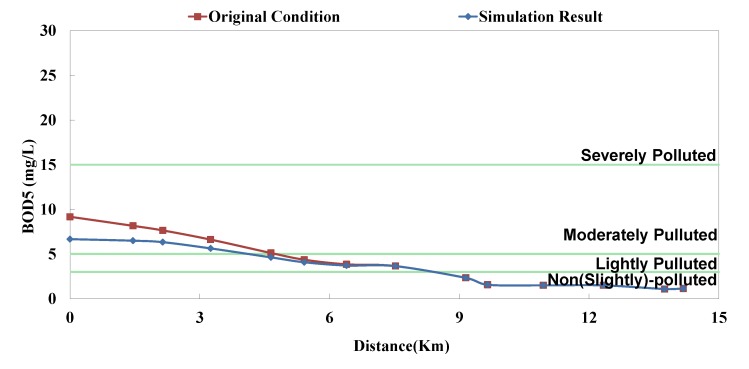
Danshui River water pollution remediation strategy simulation results—Xindian Creek BOD_5_ concentration.

**Figure 11:**
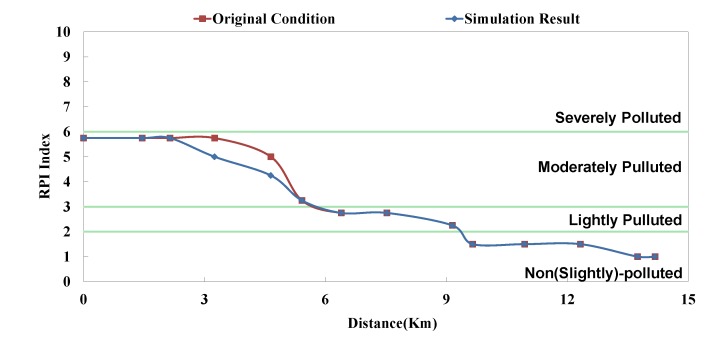
Danshui River water pollution remediation strategy simulation results—Xindian Creek RPI.

**Figure 12:**
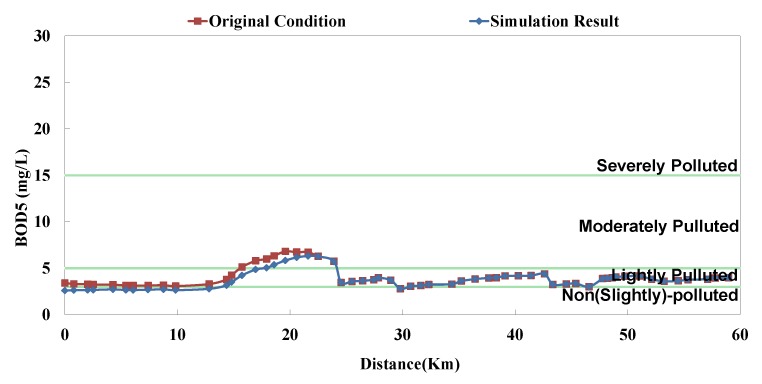
Danshui River water pollution remediation strategy simulation results—Keelung River BOD_5_ concentration.

**Figure 13:**
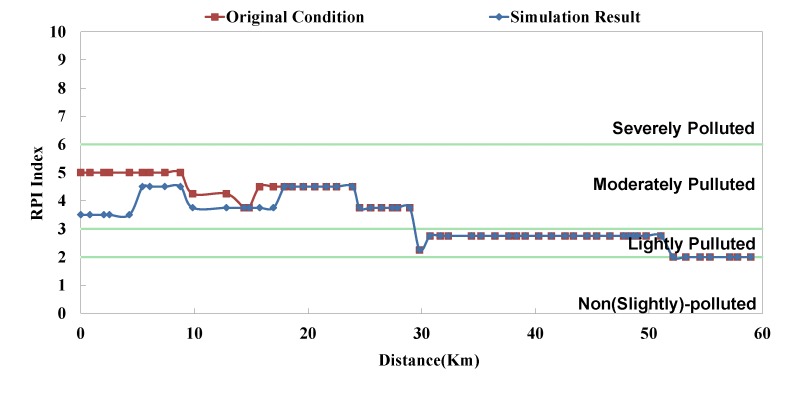
Danshui River water pollution remediation strategy simulation results—Keelung River RPI.

The analyzed optimal solution can be determined according to the impact factor and the remaining capacity of system; the result is shown in Table 4. The “Current Condition” in Table 4 means actual operation percentage compared to design flow rate. This study assumes that there are 2 strategies in each facility (*i.e.*, 0% and 100%), so the priority of each facility can be influenced not only by its capacity but also other facilities’. Although some facilities rank high, they are limited by the remaining capacity; in other words, despite of the high influence coefficient, they cannot be switched on (100%). Some facilities rank rather low, but due to sufficient remaining capacity, they are switched on (100%). The model simulation result is displayed as BOD_5_ concentration and RPI, as shown in Figure 8, Figure 9, Figure 10, Figure 11, Figure 12 and Figure 13. The “existing circumstance” refers to the average operation amount of various facilities in 2012. The figure shows the RPI under the existing operating strategy, given that the severe pollution length in the middle reach of the Dahan River is 18 km, while other reaches are below moderate pollution. The model simulation result of this study shows that the length of severe pollution in the middle reach of the Dahan River can be shortened to about 5 km. It is the basin with the most significant effectiveness; whereas there is slight water quality improvement effectiveness on the Xindian Creek and the Keelung River. 

**Table 4:** Optimal solution of optimization model.

Rank	Faculity	Creek	Current Condition	Simulation Result	Rank	Faculity	Creek	Current Condition	Simulation Result
1	X	Dahan	10%	100%	17	A	Xindian	31%	0%
2	I	Dahan	24%	100%	18	M	Dahan	26%	0%
3	Q	Dahan	100%	100%	19	Y	Dahan	68%	100%
4	AC	Dahan	56%	100%	20	U	Dahan	0%	0%
5	T	Keelung	42%	100%	21	F	Dahan	5%	0%
6	AA	Dahan	41%	100%	22	V	Keelung	47%	100%
7	L	Dahan	12%	100%	23	Z	Dahan	100%	100%
8	B	Xindian	35%	0%	24	S	Xindian	26%	0%
9	E	Dahan	16%	100%	25	O	Dahan	100%	100%
10	R	Xindian	77%	0%	26	J	Keelung	47%	0%
11	P	Dahan	100%	100%	27	G	Dahan	19%	0%
12	H	Dahan	64%	0%	28	N	Dahan	22%	0%
13	D	Xindian	83%	0%	29	AE	Xindian	5%	0%
14	C	Xindian	19%	0%	30	AD	Keelung	49%	0%
15	K	Dahan	0%	0%	31	W	Keelung	23%	100%
16	AB	Keelung	38%	100%					

The optimal strategy combination of this study and current condition are compared in Table 5. It is observed that after optimization, the facility operation amount is less than the current condition, but the total sewage treatment capacity is slightly higher than the current condition. In other words, the treatment capacity tends to concentrate, and the basin average water quality is apparently improved. Considering the changes in treatment capacity distribution, the optimized treatment capacity of the Dahan River is much higher than the current condition, that of the Xindian Creek is much lower than the current condition, and that of the Keelung River is slightly lower than the current condition. The simulation result is compared with the rate of household connection of various basins. It is found that the rate of household connection of the Dahan River basin is the lowest, suggesting that the domestic sewage in the reach has a high impact on the river water quality. Therefore, the highest effectiveness can be obtained by implementing remediation strategy on the reach. As the Keelung River basin has a higher rate of household connection, there are fewer optimized facilities in the reach. Moreover, the domestic sewage influences the river slightly, the reach pollution condition is slighter than the Dahan River, and the water quality improvement effectiveness is low. The Xindian Creek is a special case. Figure 6, Figure 7, Figure 8, Figure 9, Figure 10 and Figure 11 indicate that the simulation result is similar to the current condition. However, Table 5 shows that the sewage operation capacity and the quantity of facilities switched on in the simulation results are reduced greatly. In other words, although the rate of household connection of the Xindian Creek basin is relatively low, the river base discharge is large, and the start-stop of facilities influences the river water quality slightly.

**Table 5:** Comparison between optimal strategy combination and current condition.

Tributary	Q(CMD)		Number of Operating Facilities		Rate of Household Connection
	Current Condition	Simulation Result	Current Condition	Simulation Result	
Dahan River	474,842	796,700	28	23	25.1%
Hsindian Creek	298,251	39,700	11	4	36.7%
Keelung River	237,378	203,080	14	12	69.1%
Total	1,010,471	1,039,480	53	39	--

## 5. Conclusions

### 5.1. Combination of Taguchi Method into the Optimization Model

This study incorporated the Taguchi method into the optimization model for solution. According to the sequence of influence of parameters on the system and the limitation of optimization model system capacity, the optimal solution of the optimization problem was determined. The model computing time was shortened greatly.

### 5.2. Model Result

The facility influence weights were obtained using the Taguchi method, and the optimum combination was screened according to the remaining capacity. The result suggested that the simulation result of this study is better than the current condition, and the Dahan River is the basin with the highest water quality improvement effectiveness. Under the optimal strategy of this study, the severe pollution length was shortened from about 18 km to about 5 km.

### 5.3. Relationship between Model Result and Household Connection

According to the comparison between simulation result and the rate of household connection of basins, the basin water quality improvement effectiveness was proportional to the river pollution condition, and inversely proportional to the rate of household connection of basins.

### 5.4. Suggestions for Future Research

This study used two operating strategies for strategy configuration. Future studies can increase the operating strategies, from the On (100%) and Off (0%) strategies to operating altitudes of different openings, allowing the facilities to be used more flexibly and effectively.
